# Supplementary figures and images for: Superoxide dismutase activity in tear fluid and blood of patients and mouse model of amyotrophic lateral sclerosis: a pilot study
Source: PeerJ. 2025 Jul 1;13:e19623. doi: 10.7717/peerj.19623 (PMC12227013; doi:10.7717/peerj.19623)

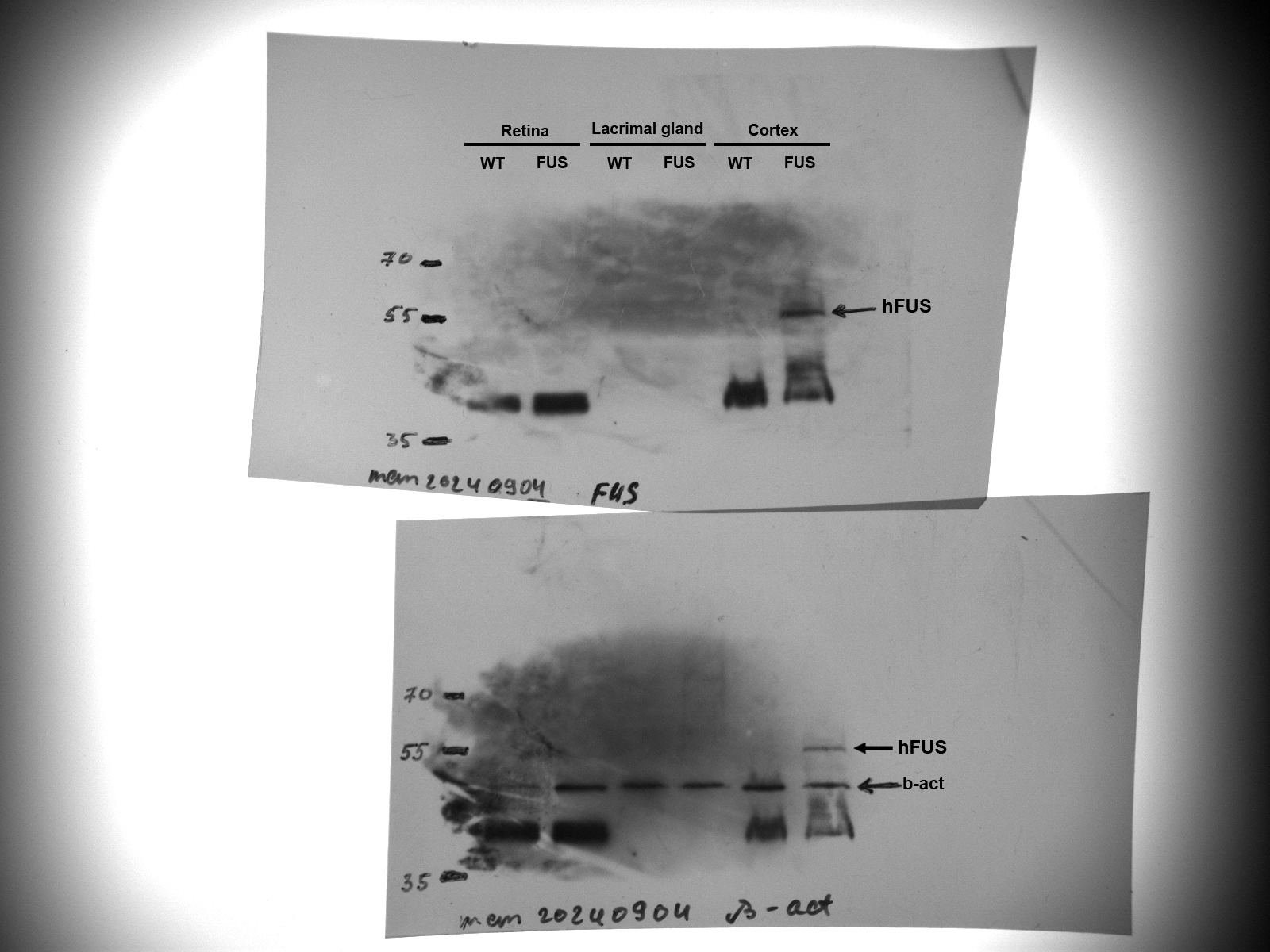

Supplement: Supplemental Information 2 [file peerj-13-19623-s002.tif]
