# Supplementary material for: Superoxide dismutase activity in tear fluid and blood of patients and mouse model of amyotrophic lateral sclerosis: a pilot study
Source: PeerJ. 2025 Jul 1;13:e19623. doi: 10.7717/peerj.19623 (PMC12227013; doi:10.7717/peerj.19623)
Supplement: Supplemental Information 3 [file peerj-13-19623-s003.docx]

STROBE Statement—checklist of items that should be included in reports of observational studies

|  | Item No | Recommendation |
| --- | --- | --- |
| **Title and abstract** | 1 | (*a*) Indicate the study’s design with a commonly used term in the title or the abstract  Pilot study as stated in the title on page 1. |
|  |  | (*b*) Provide in the abstract an informative and balanced summary of what was done and what was found  Provided in Abstract on page 1. |
| Introduction | | |
| Background/rationale | 2 | Explain the scientific background and rationale for the investigation being reported  Included in the Introduction on pages 2 and 3. |
| Objectives | 3 | State specific objectives, including any prespecified hypotheses  Included in the Introduction on page 3. |
| Methods | | |
| Study design | 4 | Present key elements of study design early in the paper  Included in the Materials and Methods on page 3 and 4. |
| Setting | 5 | Describe the setting, locations, and relevant dates, including periods of recruitment, exposure, follow-up, and data collection  Included in the Materials and Methods on pages 3 and 4. |
| Participants | 6 | (*a*) *Cohort study*—Give the eligibility criteria, and the sources and methods of selection of participants. Describe methods of follow-up  *Case-control study*—Give the eligibility criteria, and the sources and methods of case ascertainment and control selection. Give the rationale for the choice of cases and controls  *Cross-sectional study*—Give the eligibility criteria, and the sources and methods of selection of participants  Included in the Materials and Methods on pages 3 and 4. |
|  |  | (*b*) *Cohort study*—For matched studies, give matching criteria and number of exposed and unexposed  *Case-control study*—For matched studies, give matching criteria and the number of controls per case  Not applicable. |
| Variables | 7 | Clearly define all outcomes, exposures, predictors, potential confounders, and effect modifiers. Give diagnostic criteria, if applicable  Included in the Materials and Methods on pages 3 and 4. |
| Data sources/ measurement | 8* | For each variable of interest, give sources of data and details of methods of assessment (measurement). Describe comparability of assessment methods if there is more than one group  Included in the Materials and Methods on pages 3, 4 and 5 and in the figure legends. |
| Bias | 9 | Describe any efforts to address potential sources of bias  Suspected cases were excluded from the analysis, and all healthy volunteers, with a similar distribution of gender and age, were randomly assigned to groups to minimize bias and confounding. |
| Study size | 10 | Explain how the study size was arrived at  Samples were collected from patients who were observed in the clinic over a one-year period. |
| Quantitative variables | 11 | Explain how quantitative variables were handled in the analyses. If applicable, describe which groupings were chosen and why  Included in the Materials and Methods on pages 3, 4 and 5 and in the figure legends. |
| Statistical methods | 12 | (*a*) Describe all statistical methods, including those used to control for confounding  Included in the Materials and Methods on pages 3, 4 and 5 and in the figure legends. |
|  |  | (*b*) Describe any methods used to examine subgroups and interactions  Included in the Materials and Methods on pages 3, 4 and 5 and in the figure legends. |
|  |  | (*c*) Explain how missing data were addressed  There were no missing data. |
|  |  | (*d*) *Cohort study*—If applicable, explain how loss to follow-up was addressed  *Case-control study*—If applicable, explain how matching of cases and controls was addressed  *Cross-sectional study*—If applicable, describe analytical methods taking account of sampling strategy  Included in the Materials and Methods on pages 3, 4 and 5 and in the figure legends. |
|  |  | (*e*) Describe any sensitivity analyses  Not applicable. |

Continued on next page

| Results | | |
| --- | --- | --- |
| Participants | 13* | (a) Report numbers of individuals at each stage of study—eg numbers potentially eligible, examined for eligibility, confirmed eligible, included in the study, completing follow-up, and analysed  Included in the Materials and Methods on pages 3, 4 and 5 and in the figure legends. |
|  |  | (b) Give reasons for non-participation at each stage  Not applicable. |
|  |  | (c) Consider use of a flow diagram  Not required. |
| Descriptive data | 14* | (a) Give characteristics of study participants (eg demographic, clinical, social) and information on exposures and potential confounders  Included in the Materials and Methods on pages 3, 4 and in the supplemental files. |
|  |  | (b) Indicate number of participants with missing data for each variable of interest  Not applicable. |
|  |  | (c) *Cohort study*—Summarise follow-up time (eg, average and total amount)  Not applicable. |
| Outcome data | 15* | *Cohort study*—Report numbers of outcome events or summary measures over time |
|  |  | *Case-control study—*Report numbers in each exposure category, or summary measures of exposure |
|  |  | *Cross-sectional study—*Report numbers of outcome events or summary measures  Not applicable. |
| Main results | 16 | (*a*) Give unadjusted estimates and, if applicable, confounder-adjusted estimates and their precision (eg, 95% confidence interval). Make clear which confounders were adjusted for and why they were included |
|  |  | (*b*) Report category boundaries when continuous variables were categorized |
|  |  | (*c*) If relevant, consider translating estimates of relative risk into absolute risk for a meaningful time period  Not applicable. |
| Other analyses | 17 | Report other analyses done—eg analyses of subgroups and interactions, and sensitivity analyses  Not applicable. |
| Discussion | | |
| Key results | 18 | Summarise key results with reference to study objectives  Included in the Discussion on pages 6 and 7. |
| Limitations | 19 | Discuss limitations of the study, taking into account sources of potential bias or imprecision. Discuss both direction and magnitude of any potential bias  Included in the Discussion on pages 6 and 7. |
| Interpretation | 20 | Give a cautious overall interpretation of results considering objectives, limitations, multiplicity of analyses, results from similar studies, and other relevant evidence  Included in the Discussion on pages 6 and 7. |
| Generalisability | 21 | Discuss the generalisability (external validity) of the study results  Included in the Discussion on pages 6 and 7. |
| Other information | | |
| Funding | 22 | Give the source of funding and the role of the funders for the present study and, if applicable, for the original study on which the present article is based  Provided in the text on page 8. |

*Give information separately for cases and controls in case-control studies and, if applicable, for exposed and unexposed groups in cohort and cross-sectional studies.

**Note:** An Explanation and Elaboration article discusses each checklist item and gives methodological background and published examples of transparent reporting. The STROBE checklist is best used in conjunction with this article (freely available on the Web sites of PLoS Medicine at http://www.plosmedicine.org/, Annals of Internal Medicine at http://www.annals.org/, and Epidemiology at http://www.epidem.com/). Information on the STROBE Initiative is available at www.strobe-statement.org.
